# Bullous pemphigoid associated with vildagliptin in an elderly adult with diabetes mellitus. First case in Peru

**DOI:** 10.17843/rpmesp.2025.421.13871

**Published:** 2025-02-17

**Authors:** Marcio Concepción-Zavaleta, Cristian D. Armas, Juan Eduardo Quiroz-Aldave, Janet Ángeles-Zavaleta, María del Carmen Durand-Vásquez, José Paz-Ibarra, Janneth Y. Quispe-Meza, Luis Concepción-Urteaga

**Affiliations:** 1 Universidad Científica del Sur, Lima, Peru. Universidad Científica del Sur Lima Peru; 2 School of Medicine. National University of Trujillo, Trujillo, Peru. School of Medicine National University of Trujillo Trujillo Peru; 3 Chepén Support Hospital. Chepén, Peru. Chepén Support Hospital Chepén Peru; 4 “Abraza La Vida” Community Mental Health Center. Pueblo Nuevo, Peru. “Abraza La Vida” Community Mental Health Center Pueblo Nuevo Peru; 5 School of Medicine, National University of San Marcos, Lima, Peru. National University of San Marcos ">School of Medicine National University of San Marcos Lima Peru; 6 Cajamarca Regional Teaching Hospital. Cajamarca, Peru. Cajamarca Regional Teaching Hospital Cajamarca Peru; 7 Trujillo Regional Teaching Hospital. Trujillo, Peru. Trujillo Regional Teaching Hospital Trujillo Peru

**Keywords:** Bullous Pemphigoid, Dipeptidyl-Peptidase IV Inhibitors, Vildagliptin, Drug-Related Side Effects and Adverse Reactions, Case Reports

## Abstract

Vildagliptin, a dipeptidyl peptidase-4 inhibitor (DPP-4i) used in the treatment of type 2 diabetes mellitus (DM2), stands out for its safety in older adults. However, it is associated with adverse reactions, such as bullous pemphigoid (BP), although no cases have been documented in Peru to date. We report the case of a 76-year-old male patient with hypertension and DM2 who started treatment with vildagliptin and metformin. After one month, he presented pruritic, bullous and ulcerated skin lesions, leading to the diagnosis of BP. BP, associated with DPP-4i, mainly affects older adults, manifesting on average about 9 months after the start of treatment. Its diagnosis is based on clinical criteria, histopathology and immunofluorescence. Treatment includes discontinuation of the drug and the use of corticosteroids. Vildagliptin, although safe and effective, can cause BP, which requires timely diagnosis and treatment due to its high mortality.

## INTRODUCTION

Vildagliptin, a dipeptidyl peptidase-4 inhibitor (DPP-4i), is used to treat type 2 diabetes mellitus (DM2) because it increases the concentrations of incretins, which stimulate the release of insulin and reduce glucagon secretion in a glucose-dependent manner. It can be administered as monotherapy or in combination with other antidiabetic drugs ^1^. Its effectiveness in glycemic control, its low risk of hypoglycemia, and its neutral effect on body weight make it a safe therapeutic option in the elderly population ^1^.

Although generally well tolerated ^2^, vildagliptin can cause mild adverse reactions such as headaches, nasopharyngitis, back pain and dizziness, as well as rare but serious events such as necrotizing pancreatitis and hypersensitivity reactions ^3^. An increased risk of adverse skin effects has been reported with the use of DPP-4i ^4^, including a significant association with bullous pemphigoid (BP) in several systematic reviews ^(4-6)^, which suggests a class effect ^7^. However, the results of these reviews may vary regarding which is the drug with the strongest association, pointing to vildagliptin ^6^, linagliptin ^8^, or sitagliptin ^4^. In addition, North American, European and Asian pharmacovigilance reports highlight the strong association between DPP-4i and BP ^9^^-^^13^.

This study aimed to report the first case registered in Peru of adverse drug reactions associated with vildagliptin in an elderly adult, detailing its manifestations and evolution to contribute to pharmacovigilance and increase knowledge about the safety of vildagliptin.

## CASE REPORT

We present the case of a 76-year-old male patient with no relevant family history, diagnosed with arterial hypertension and being treated with diltiazem 60 mg once a day, as well as DM2 diagnosed 40 years ago, and being treated with vildagliptin 50 mg and metformin 1000 mg twice a day for the last month, after which he reports noticing the appearance of itchy skin lesions on his arms, legs and head. Two months later, due to the suspicion of an adverse skin reaction associated with vildagliptin, the treatment was modified, changing to glimepiride 2 mg and metformin 1000 mg once a day. However, one month after this change, the lesions persisted. In view of this situation, the patient was hospitalized to complete the diagnosis and begin treatment.

**Figure 1:**
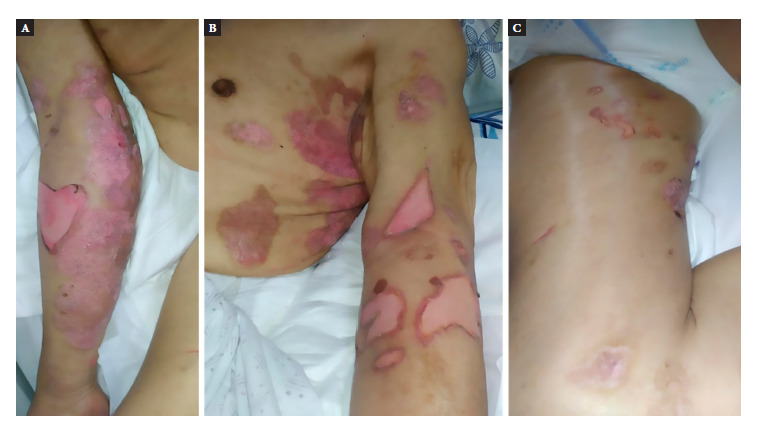
Lesions on the patient’s upper limbs (A and B) and lower limbs consistent with bullous pemphigoid (C).

The physical examination showed a weight of 54 kg, height of 160 cm, blood pressure of 100/60 mm Hg, heart rate of 102 beats per minute and respiratory rate of 16 breaths/minute. Multiple blistering and ulcerative lesions were found, with irregular edges, symmetrical distribution, with surrounding erythema and a positive Nikolsky sign, located on the upper limbs (Figure 1 A and B), lower limbs (Figure 1 C) and scalp. The rest of the physical examination did not show significant alterations.

**Figure 2:**
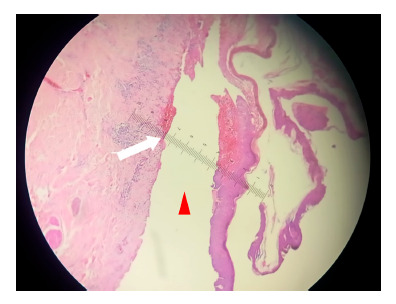
Microphotograph of skin biopsy at 40x, using hematoxylin-eosin. Note the subepidermal vesicle (red arrowhead) under which there is an infiltrate of lymphocytes with some eosinophils (white arrow).

Laboratory results showed a leukocyte count of 16,000 cells/cc, with 4% blasts, hemoglobin 12 g/dL, platelets 210,000 cells/cc, creatinine 0.8 g/dL, glucose 102 mg/dL, aspartate aminotransferase 32 U/L, alanine aminotransferase 28 U/L and C-reactive protein 80 mg/L. The urine test, urine culture and chest X-ray showed no abnormalities. Due to the initial diagnosis of sepsis with a skin focus, antibiotic treatment started with ceftriaxone 2 g intravenously once a day and clindamycin 600 mg intravenously three times a day for two weeks. A biopsy of the skin lesions was carried out, which confirmed the presence of BP (Figure 2).

**Figure 3:**
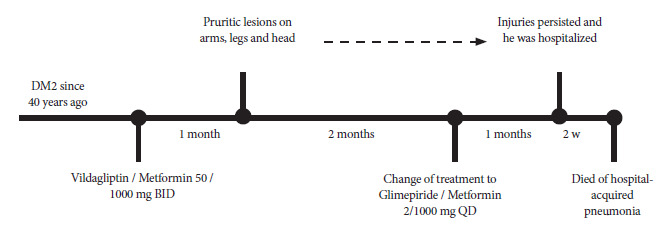
Timeline showing the history of the patient's disease from onset to death. BID: twice daily; DM2: diabetes mellitus type 2; w: weeks; QD: once daily.

After completing treatment, pulses of methylprednisolone were planned to managed BP, however, unfortunately, the patient died two weeks later due to respiratory failure secondary to hospital-acquired pneumonia. The most relevant patient data is summarized in Figure 3.

## DISCUSSION

BP is a rare autoimmune skin disease ^5^, the incidence of which has increased in recent decades, partly attributed to the aging of the population ^14^. More than 60 drugs have been identified as associated with BP, including antibiotics, diuretics, antihypertensive medication, anti-TNF-α drugs, vaccines, immune checkpoint inhibitors against programmed death-1 and others. However, the highest risk has been reported with DPP-4i ^14^. The period between the start of DPP-4i and BP is nine months on average ^15^, but can be more than a year, which suggests that DPP-4i-associated BP could be a disease exacerbated by the treatment, rather than an adverse reaction ^14^. It has been estimated that one in four patients with BP die each year ^16^. When it comes to the patient we report on, the time elapsed from the start of therapy with DPP-4i to the appearance of BP was shorter than is usually reported.

Although its pathogenesis is not fully understood ^5^, it is known that there is autoimmune activity against two hemidesmosomal components: BP180, also known as collagen XVII, and BP230, also known as dystonin-e, present in the keratinocytes of the basement membrane of the skin, which maintain the junction between the epidermis and the dermis ^17^. BP180 is especially susceptible to these autoantibodies, with the non-collagenous extracellular and juxtamembranous domain 16 A (NC16A) being the binding site for approximately 90% of IgG-type autoantibodies ^14^^,^^18^. The role of BP230 is still controversial, and it could be associated with atypical forms of BP ^17^.

DPP-4 is expressed on the surface of different cell types, including T cells, B cells, natural killer cells and macrophages ^4^. DPP-4 activates and stimulates the proliferation of Th1, Th17, T regulators and macrophages, and regulates the synthesis of immunoglobulins by B cells ^19^. The activity of peripheral T cells specific against NC16A is predominantly Th2 type, with Th2-regulated IgG4 autoantibodies and Th1-regulated IgG1 autoantibodies found in patients with BP ^20^. In addition, IgE autoantibodies have been identified mainly against the NC16A domain of BP180 ^17^. The binding of autoantibodies to their epitopes activates complement (C3a and C5a), induces the chemotaxis of neutrophils and eosinophils, the degranulation of mast cells and the release of proteolytic enzymes, and finally, the degradation of the dermal-epidermal junction and the formation of blisters ^14^^,^^17^.

Genetic susceptibility to BP involves the presence of the HLA-DQB1*0301 allele of MHC II ^17^, as well as the HLA-DRB1*04, DRB1*1101 and DQB1*0302 alleles in Japanese patients ^21^. In addition, an association has been reported with polymorphisms in the MT-ATP8 gene ^22^, CYP2D6 ^23^ and FcγRIIIa receptors ^24^. In our case, it was not possible to study the patient’s genetic susceptibility.

Furthermore, DM2 itself could be associated with the development of BP, increasing its incidence and mortality ^8^. DPP-4i-induced BP has characteristics similar to conventional BP, but can persist for several months after discontinuation of the drug and may experience rare relapses. Non-inflammatory phenotype BP is mainly found in Japanese patients, with negative autoantibodies against BP180-NC16A, less eosinophil infiltration in peri-bullous lesions, and milder, less inflammatory skin lesions ^7^. This is probably why discontinuation of the DPP-4i has not been associated with an immediate improvement in skin lesions.

The diagnosis of BP is based on clinical characteristics, histopathology and immunofluorescence ^17^. It usually begins with a prodrome of intense pruritus for several days or months. Classic BP is characterized by the appearance of tense, serous or hemorrhagic blisters, highly pruritic, 1 to 3 cm in diameter, on erythematous skin. These are distributed symmetrically on the lower abdomen, the flexor surfaces of the legs, the inguinal and axillary areas, and rarely on the oral mucosa. Over time, the blisters turn into hypopigmented scabs without leaving scars, and the condition presents spontaneous cyclical exacerbations and remissions for several years ^17^. Eczematous urticarial or excoriated plaques or prurigo-like lesions may also appear ^14^, as in our case, on the arms, legs and head, which delayed diagnosis. The characteristics of BP are the same regardless of the use of DPP-4i ^14^. In terms of severity, skin involvement of less than 10% has been defined as mild, between 10% and 30% as moderate, and over 30% as severe ^25^.

Skin biopsy reveals subepidermal blisters with inflammation and eosinophilic infiltrates ^14^, such as those observed in the study of our patient. Direct immunofluorescence microscopy, considered the gold standard, shows linear deposits of IgG and/or complement along the basement membrane, but has low specificity. Indirect immunofluorescence microscopy on salt-split skin is recommended in all cases of suspected BP due to its high specificity. The enzyme immunoassay detects autoantibodies against BP180-NC16A and BP230, correlating with disease activity and risk of relapse. Furthermore, most patients present high levels of serum IgE and eosinophilia ^17^. One of the limitations of our case is that it was not possible to carry out immunofluorescence studies or enzyme immunoassays, due to the patient’s torpid evolution and logistical and economic difficulties.

Treatment of BP emphasizes the suspension of the triggering agent, if identified, although there is still no universally accepted therapeutic approach ^14^. In our case study, the suspension of the medication occurred at the time of the identification of the initial lesions, even though BP had not yet been diagnosed due to the non-specificity of the lesions. Topical treatment as monotherapy is recommended in mild cases, in moderate cases it can be used in isolation or in combination with systemic therapy, and in severe cases it should be combined with systemic glucocorticoids ^25^. It is suggested that glucocorticoid therapy be initiated with an equivalent prednisolone dose of 0.2-0.5 mg/kg of body weight per day, orally, with the aim of reducing inflammation and blistering. Once the formation of new lesions is stopped, the dose of systemic glucocorticoids should be gradually reduced ^25^. Regarding adjuvants, azathioprine is considered the first option, followed by mycophenolate mofetil ^25^. Systemic therapy was planned but was not started due to the patient’s poor progress and his death.

Vildagliptin, although considered a safe antidiabetic therapy in the elderly, is associated with adverse reactions such as BP. In some cases, diagnosis may be delayed due to the non-specificity of the initial lesions. The associated mortality is high, highlighting the importance of timely diagnosis and treatment to reduce the risk of complications.
