# Supplementary material for: Bullous pemphigoid associated with vildagliptin in an elderly adult with diabetes mellitus. First case in Peru
Source: Rev Peru Med Exp Salud Publica. 2025 Feb 17;42(1):97–101. doi: 10.17843/rpmesp.2025.421.13871 (PMC12176020; doi:10.17843/rpmesp.2025.421.13871)
Supplement: Translation

# Translation: Penfigoide bulloso asociado a vildagliptina en adulto mayor con diabetes mellitus. Primer caso en Perú

**Authors:** Marcio Concepción-Zavaleta, Cristian D. Armas, Juan Eduardo Quiroz-Aldave, Janet Ángeles-Zavaleta, María del Carmen Durand-Vásquez, José Paz-Ibarra, Janneth Y. Quispe-Meza, Luis Concepción-Urteaga

**Affiliations:** 1 Universidad Científica del Sur, Lima, Perú.; 2 School of Medicine. National University of Trujillo, Trujillo, Perú.; 3 Chepén Support Hospital. Chepén, Perú.; 4 Centro de Salud Mental Comunitario “Abraza La Vida”. Pueblo Nuevo, Perú.; 5 Escuela de Medicina, Universidad Nacional Mayor de San Marcos, Lima, Perú.; 6 Hospital Regional Docente de Cajamarca. Cajamarca, Perú.; 7 Hospital Regional Docente de Trujillo. Trujillo, Perú.

**Keywords:** Penfigoide Ampolloso, Inhibidores de la Dipeptidil-Peptidasa IV, Vildagliptina, Efectos Colaterales y Reacciones Adversas Relacionados con Medicamentos, Informes de Casos

## Abstract

La vildagliptina, un inhibidor de la dipeptidil peptidasa-4 (DPP-4i) utilizado en el tratamiento de la diabetes mellitus tipo 2 (DM2), se destaca por su seguridad en adultos mayores. Sin embargo, se asocia con reacciones adversas, como el penfigoide ampollar (PA), aunque hasta el momento no se han documentado casos en Perú. Se reporta un paciente varón de 76 años con hipertensión y DM2 que inició tratamiento con vildagliptina y metformina. Después de un mes, presentó lesiones cutáneas pruriginosas, ampollosas y ulceradas, conduciendo al diagnóstico de PA. El PA, asociado a DPP-4i, afecta principalmente a adultos mayores, manifestándose en promedio unos 9 meses después del inicio del tratamiento. Su diagnóstico se basa en criterios clínicos, histopatología e inmunofluorescencia. El tratamiento incluye la suspensión del fármaco y el uso de corticoides. La vildagliptina, aunque segura y efectiva, puede provocar PA, que requiere un diagnóstico y tratamiento oportuno debido a su alta mortalidad.

## INTRODUCCIÓN

La vildagliptina, un inhibidor de la dipeptidil peptidasa-4 (DPP-4i), se utiliza para tratar la diabetes mellitus tipo 2 (DM2) debido a que incrementa las concentraciones de incretinas, las cuales estimulan la liberación de insulina y reducen la secreción de glucagón de manera glucosa-dependiente. Puede administrarse en monoterapia o en combinación con otros antidiabéticos ^1^. Su eficacia en el control glicémico, su bajo riesgo de hipoglicemia, y su efecto neutral sobre el peso corporal lo convierten en una opción terapéutica segura en la población de edad avanzada ^1^.

Aunque generalmente es bien tolerada ^2^, la vildagliptina puede causar reacciones adversas leves como cefaleas, nasofaringitis, dolor de espalda y mareos, así como eventos raros pero graves como pancreatitis necrotizante y reacciones de hipersensibilidad ^3^. Se ha observado un aumento del riesgo de efectos adversos cutáneos con el uso de DPP-4i ^4^, incluida su asociación significativa con el penfigoide ampollar (PA) en varias revisiones sistemáticas ^4^^-^^6^, lo cual sugiere un efecto de clase ^7^. Sin embargo, los resultados de estas revisiones pueden variar en cuanto al medicamento con mayor asociación, señalando a la vildagliptina ^6^, linagliptina ^8^, o sitagliptina ^4^. Además, los informes de farmacovigilancia norteamericanos, europeos y asiáticos destacan la fuerte asociación entre los DPP-4i y el PA ^9^^-^^13^.

El presente estudio tiene como objetivo reportar el primer caso registrado en Perú de PA asociado a vildagliptina en un adulto mayor, detallando sus manifestaciones y evolución para contribuir a la farmacovigilancia y aumentar el conocimiento sobre la seguridad de la vildagliptina.

## REPORTE DE CASO

Paciente varón de 76 años sin antecedentes familiares contributorios, con diagnóstico de hipertensión arterial en tratamiento con diltiazem 60 mg una vez al día, así como DM2 diagnosticada 40 años atrás, y en tratamiento con vildagliptina 50 mg y metformina 1000 mg dos veces desde hace 1 mes, luego de lo cual refiere notar la aparición de lesiones cutáneas pruriginosas en brazos, piernas y cabeza. Dos meses después, debido a la sospecha de una reacción adversa cutánea asociada a vildagliptina, se decidió modificar el tratamiento, cambiando a glimepirida 2 mg y metformina 1000 mg una vez al día. Sin embargo, un mes después de dicho cambio, las lesiones persistían. Ante esta situación, el paciente fue hospitalizado para completar el diagnóstico y comenzar con el tratamiento.

En el examen físico, se evidenció peso 54 kg, talla 160 cm, presión arterial 100/60 mm Hg, frecuencia cardiaca 102 latidos por minuto y frecuencia respiratoria 16 respiraciones/minuto. Se encontraron múltiples lesiones ampollosas y ulceradas, de bordes irregulares, distribución simétrica, con eritema circundante y signo de Nikolsky positivo, localizadas en los miembros superiores (Figura 1 A y B), inferiores (Figura 1 C) y cuero cabelludo. El resto del examen físico no presentó alteraciones significativas.

**Figura 1:** Lesiones en miembros superiores (A y B) y en miembros inferiores del paciente compatibles con penfigoide ampolloso (C).

En la analítica, se encontró un recuento de leucocitos de 16,000 células/cc, con un 4% de abastonados, hemoglobina 12 g/dL, plaquetas 210,000 células/cc, creatinina 0,8 g/dL, glucosa 102 mg/dL, aspartato amino transferasa 32 U/L, alanina amino transferasa 28 U/L. y proteína C reactiva 80 mg/L. El examen de orina, el urocultivo y la radiografía de tórax no presentaron alteraciones. Debido al diagnóstico inicial de sepsis con foco dérmico, se inició tratamiento antibiótico con ceftriaxona 2 g intravenosa una vez al día y clindamicina 600 mg intravenosa tres veces al día durante dos semanas. Asimismo, se procedió a realizar una biopsia de las lesiones cutáneas, cuyo resultado histopatológico (Figura 2) confirmó la presencia de PA.

**Figura 2:** Microfotografía de biopsia de piel a 40x, usando hematoxilina-eo-sina. Obsérvese vesícula subepidérmica (cabeza de flecha roja) debajo de la cual hay infiltrado de linfocitos con algunos eosinófilos (flecha blanca).

Luego de completar el tratamiento, se planificó administrar pulsos con metilprednisolona para el manejo del PA, sin embargo, lamentablemente, el paciente falleció dos semanas después debido a insuficiencia respiratoria secundaria a neumonía intrahospitalaria. Se resumen los datos más relevantes del paciente en la Figura 3.

**Figura 3:** Línea del tiempo que muestra la historia de la enfermedad del paciente desde su inicio hasta su deceso. BID: dos veces al día; DM2: diabetes mellitus tipo 2; sem: semanas; QD: una vez al día.

## DISCUSIÓN

El PA es una enfermedad cutánea autoinmune rara ^5^, cuya incidencia ha aumentado en las últimas décadas, atribuido en parte al envejecimiento de la población ^14^. Se han identificado más de 60 fármacos asociados con PA, entre ellos antibióticos, diuréticos, antihipertensivos, fármacos anti-TNF-α, vacunas, inhibidores de los *checkpoints* inmunes contra la muerte programada-1 y otros. Sin embargo, el riesgo más alto se observa con los DPP-4i ^14^. El periodo entre el inicio de los DPP-4i y el PA es de nueve meses en promedio ^15^, pero puede llegar a más de un año, lo que sugiere que el PA asociada a DPP-4i podría ser una enfermedad exacerbada por el tratamiento, en vez de una reacción adversa ^14^. Se ha estimado que uno de cada cuatro pacientes con PA fallece anualmente ^16^. En el paciente que reportamos el tiempo transcurrido desde el inicio de la terapia con DPP-4i hasta la aparición del PA fue más corto que lo habitualmente descrito.

Aunque su patogénesis no se comprende completamente ^5^, se sabe que en este trastorno, existe actividad autoinmunitaria contra dos componentes hemidesmosomales: BP180, también conocido como colágeno XVII, y BP230, también conocido como distonina-e, presentes en los queratinocitos de la membrana basal de la piel, que mantienen la unión entre la epidermis y la dermis ^17^. BP180, es especialmente susceptible a estos autoanticuerpos, siendo el dominio no colagenoso extracelular y yuxtamembranoso 16 A (NC16A) el sitio de unión para aproximadamente el 90% de los autoanticuerpos tipo IgG ^14^^,^^18^. El rol de la BP230 aún es controversial, y podría estar asociada con formas atípicas de PA ^17^.

DPP-4 está expresado en la superficie de diferentes tipos de células, incluyendo células T, células B, linfocitos asesinos naturales y macrófagos ^4^. DPP-4 activa y estimula la proliferación de Th1, Th17, T reguladores, y macrófagos, y regula la síntesis de inmunoglobulinas de las células B ^19^. La actividad de las células T periféricas específicas contra el NC16A es predominantemente de tipo Th2, encontrando autoanticuerpos IgG4 regulados por Th2 y autoanticuerpos IgG1 regulados por Th1 en pacientes con PA ^20^. Además, se han identificado autoanticuerpos de clase IgE principalmente contra el dominio NC16A del BP180 ^17^. La unión de los autoanticuerpos a sus epítopos activa el complemento (C3a y C5a), induce la quimiotaxis de neutrófilos y eosinófilos, la degranulación de mastocitos y liberación de enzimas proteolíticas, y finalmente, la degradación de la unión dermoepidérmica y la formación de ampollas ^14^^,^^17^.

La susceptibilidad genética en el PA implica la presencia del alelo HLA-DQB1*0301 del MHC II ^17^, así como los alelos HLA-DRB1*04, DRB1*1101 y DQB1*0302 en pacientes japoneses ^21^. Además, se ha observado asociación con polimorfismos en el gen MT-ATP8 ^22^, CYP2D6 ^23^ y receptores FcγRIIIa ^24^. En nuestro caso no se pudo estudiar la susceptibilidad genética del paciente.

Además, la DM2 por sí misma podría estar asociada con el desarrollo de PA, aumentando su incidencia y mortalidad ^8^. El PA inducido por DPP-4i, presenta características similares al PA convencional, pero puede persistir durante varios meses después de la discontinuación del fármaco y podría experimentar recaídas poco frecuentes. El PA de fenotipo no inflamatorio se observa principalmente en pacientes japoneses, con autoanticuerpos negativos contra BP180-NC16A, menor infiltración de eosinófilos en las lesiones periampollosas, y lesiones cutáneas más leves, y menos inflamatorias ^7^. Es probable que esta sea la razón por la cual la descontinuación del DPP-4i no se haya asociado con una mejora inmediata en las lesiones cutáneas.

El diagnóstico de PA se basa en características clínicas, histopatología e inmunofluorescencia ^17^. Suele iniciar con un pródromo de prurito intenso por varios días o meses. El PA clásico se caracteriza por la aparición de ampollas tensas, serosas o hemorrágicas, altamente pruriginosas, de 1 a 3 cm de diámetro, sobre una piel eritematosa. Estas se distribuyen simétricamente en el abdomen inferior, las superficies flexoras de las piernas, la zona inguinal y axilar, y raramente en la mucosa oral. Con el tiempo, las ampollas se transforman en costras hipopigmentadas sin dejar cicatrices, y el cuadro presenta exacerbaciones y remisiones cíclicas espontáneas durante varios años ^17^. También pueden aparecer placas eccematosas urticariales o excoriadas, o lesiones similares a prurigo ^14^, como en nuestro caso, localizándose en brazos, piernas y cabeza, lo cual retrasó el diagnóstico. Las características del PA son las mismas independientemente del uso de DPP-4i ^14^. En cuanto a severidad, se ha definido como leve a una afectación cutánea menor al 10%, moderada entre el 10% y el 30%, y grave por encima del 30% ^25^.

La biopsia cutánea revela ampollas subepidérmicas con inflamación e infiltrados eosinofílicos ^14^, tales como los observados en el estudio de nuestro paciente. La microscopía de inmunofluorescencia directa, considerada el estándar de oro, muestra depósitos lineales de IgG y/o complemento a lo largo de la membrana basal, pero tiene poca especificidad. La microscopía de inmunofluorescencia indirecta en piel dividida con sal se recomienda en todos los casos sospechosos de PA por su alta especificidad. El ensayo inmunoenzimático detecta autoanticuerpos contra BP180-NC16A y BP230, correlacionando con la actividad de la enfermedad y el riesgo de recaída. Además, la mayoría de los pacientes presenta altos niveles de IgE sérica y eosinofilia ^17^. Una de las limitaciones de nuestro caso es que no fue posible realizar estudios de inmunofluorescencia ni ensayo inmunoenzimático, debido a la evolución tórpida del paciente y a dificultades logísticas y económicas.

El tratamiento del PA enfatiza la suspensión del agente desencadenante, si se identifica, aunque aún no existe un enfoque terapéutico universalmente aceptado ^14^. En nuestro caso de estudio, la suspensión del medicamento ocurrió al momento de la identificación de las lesiones iniciales incluso a pesar de aún no haber diagnosticado PA debido a la inespecificidad de estas. El tratamiento tópico en monoterapia se recomienda en casos leves, mientras que en los casos moderados puede utilizarse de forma aislada o en combinación con terapia sistémica, y en los casos graves debe combinarse con glucocorticoides sistémicos ^25^. Se sugiere iniciar la terapia con glucocorticoides con una dosis de prednisolona equivalente de 0,2-0,5 mg/kg de peso corporal por día, por vía oral, con el objetivo de reducir la inflamación y la formación de ampollas. Una vez que se detiene la formación de nuevas lesiones, se debe reducir gradualmente la dosis de glucocorticoides sistémicos ^25^. Respecto a los adyuvantes, la azatioprina se considera la primera opción, seguida del micofenolato de mofetilo ^25^. A pesar de la planificación de inicio de la terapia sistémica, la mala evolución del paciente y su deceso impidieron su aplicación.

La vildagliptina, aunque considerada una terapia antidiabética segura en ancianos, se asocia con reacciones adversas, como el PA. En algunos casos, su diagnóstico puede retrasarse por la inespecificidad de las lesiones iniciales. La mortalidad asociada es alta, resaltando la importancia del diagnóstico y tratamiento oportunos para reducir el riesgo de complicaciones.
